# Efficient extracellular recombinant production and purification of a *Bacillus* cyclodextrin glucanotransferase in *Escherichia coli*

**DOI:** 10.1186/s12934-017-0701-1

**Published:** 2017-05-19

**Authors:** Christian Sonnendecker, Ren Wei, Elisabeth Kurze, Jinpeng Wang, Thorsten Oeser, Wolfgang Zimmermann

**Affiliations:** 1 Institute of Biochemistry, Department of Microbiology and Bioprocess Technology, Leipzig University, Johannisallee 23, 04103 Leipzig, Germany; 2 State Key Laboratory of Food Science and Technology, Jiangnan University, Wuxi, 214122 Jiangsu China

**Keywords:** *Bacillus* sp. G825-6, *Escherichia coli*, Cyclodextrin glucanotransferase, Enzyme production, Heterologous expression, Signal peptides, Protein secretion

## Abstract

**Background:**

Cyclodextrin glucanotransferases (CGTases) catalyze the synthesis of cyclodextrins, cyclic oligosaccharides composed of glucose monomers that find applications in the pharmaceutical, food, and cosmetic industries. An economic application of these industrially important enzymes requires their efficient production and recovery. In this study, the effect of Sec-type signal peptides on the recombinant expression of a CGTase derived from *Bacillus* sp. G825-6 was investigated in *Escherichia coli* BL21(DE3) using a codon-adapted gene. In addition, a novel purification method for the CGTase using starch adsorption was developed.

**Results:**

Expression vectors encoding N-terminal PelB, DacD, and the native *Bacillus* sp. G825-6 CGTase signal peptides (SP) were constructed for the recombinant CGTase. With the DacD SP derived from *E. coli,* a 3.9- and 3.1-fold increase in total enzyme activity was obtained compared to using the PelB and the native CGTase SP, respectively. DacD enabled a 7.3-fold increase of activity in the extracellular fraction after induction for 24 h compared to the native CGTase SP. After induction for 48 h, 75% of the total activity was detected in the extracellular fraction. By a batch wise adsorption to starch, the extracellular produced CGTase could be purified to homogeneity with a yield of 46.5% and a specific activity of 1637 U/mg.

**Conclusions:**

The signal peptide DacD promoted the high-level heterologous extracellular expression of a recombinant CGTase from *Bacillus* sp. G825-6 with a pET20b(+) vector in *E. coli* BL21(DE3). A protocol based on starch adsorption enabled a fast and efficient purification of the recombinant enzyme.

## Background

Cyclodextrins (CD) are cyclic oligosaccharides derived from starch. They are synthesized industrially by cyclodextrin glucanotransferases (CGTases, EC 2.4.1.19) [1, 2]. The smallest naturally occurring CD produced by CGTases is cyclomaltohexaose (CD6) composed of six glucose monomers [3]. Their property to form reversible complexes with guest molecules has enabled the application of CD_6_, CD_7_, and CD_8_ in many industrial areas where their demand is still growing. Larger CD, which could find interesting novel applications, have not reached the market yet since efficient processes for their production and purification are still not available [4]. CGTases form a range of CD of different ring sizes with CD_6_–CD_8_ as major and larger CD as minor products [5]. In addition to the production of CD, CGTases also find applications e.g. as anti-staling agents in the bakery industry or for the glycosylation of biomolecules [6, 7].

Cyclodextrin glucanotransferases are often produced using the original host organism or recombinantly in *Escherichia coli, Bacillus subtilis* and *Bacillus megaterium* [8, 9]. In these bacterial expression systems, a codon usage adaption for the respective expression host could efficiently increase the yield of the recombinant CGTase [8–10]. In *E. coli*, recombinant proteins are translocated into the extracellular space only to a limited extent [11, 12]. The recombinant extracellular expression of CGTases in *E. coli* has however the advantage of a simplified low-cost downstream process [13–16]. The addition of divalent metal cations, glycine or Triton X100 has been described to promote the extracellular expression of recombinant CGTase in *E. coli* [15, 17]. A further strategy is the use of signal peptides (SP) to promote the translocation of the recombinant enzyme. The recombinant SP PelB [18], OmpA [14], and SP derived from the host strains [13] have been shown to facilitate the extracellular expression of CGTases in *E. coli*. The extracellular expression of the *Bacillus* sp. G1 CGTase with the native G1-CGTase SP in *E. coli* resulted in a yield of 62.3% of the total activity in the culture medium [13]. A CGTase gene from *Paenibacillus macerans* JFB05-01 was cloned into the pET20b(+) vector with the SP OmpA [14]. Using a fed-batch protein expression strategy, up to 79% of the total activity was detected in the culture medium.

In this study, we compared the effect of PelB, DacD, and the native *Bacillus* sp. G825-6 CGTase SP on the extracellular expression of recombinant CGTase in *E. coli.* In addition, we showed that the extracellular expressed CGTase could be efficiently purified by batch wise adsorption to starch in a single step.

## Methods

### Construction of expression vectors

The codon-optimized gene coding for the *Bacillus* sp. G825-6 CGTase (*cgtS*) was cloned into pET20b(+) as described before [19, 20] and enabled the production of a recombinant CGTase with a N-terminal PelB SP. The gene encoding the DacD SP was synthesized by Life Technologies (Carlsbad, CA, USA) and cloned into the pET20b(+):*cgtS* vector via the restriction sites *Nde*I and *Bam*HI. Originally, *dacD* describes the gene encoding d-alanyl-d-alanine carboxypeptidase of *E. coli.* In this study, we refer only to the corresponding SP of DacD. The gene encoding for the DacD SP was introduced via the restriction sites *Nde*I and *Bam*HI to replace the gene for PelB SP in the pET-20b(+) vector. A construct without any SP was prepared by eliminating the *pelB* leader sequence using the *Nde*I restriction site.

### Recombinant protein expression

Lysogeny broth (LB) medium containing 100 µg/ml ampicillin was used to grow *E. coli* at 37 °C. Flasks with 250 ml medium were inoculated with overnight cultures to an optical density of 0.1 at 600 nm (OD_600_). Recombinant protein expression was induced with isopropyl-β-d-thiogalactopyranoside (IPTG) at a final concentration of 0.5 mM at an OD_600_ of 0.6. After induction, cultures were further incubated for up to 48 h at 25 °C under vigorous shaking.

### Cell fractionation

Aliquots (2 ml) were collected at different incubation times and separated into extracellular and cytoplasmic fractions with the PeriPreps™ Periplasting Kit (Epicentre, Madison, WI, USA).

### Protein quantification

Protein concentration was measured using the Bradford method with Rotiquant according to the supplier manual (Carl Roth, Karlsruhe, Germany). The assay was performed in a 96-well microplate format with a double determination at 450 and 590 nm [21]. Bovine serum albumin was used for calibration.

### Starch-degrading activity assay

One unit of starch-degrading activity was defined as the amount of CGTase that degrades 1 mg starch in 10 min at pH 8.5 and 50 °C [22]. The assay was performed as described previously [23] with the following modifications: starch solution (12 g/l) was prepared with CGTase buffer (25 mM Tris–HCl, 10 mM KCl, 10 mM MgCl_2_, pH 8.5). Enzyme solution (20 µl) was added to 100 µl of the starch solution at 50 °C.

### Purification of the recombinant CGTase by starch adsorption

A soluble starch solution (50 g/l) was prepared in CGTase buffer. The solution was boiled and immediately chilled to 4 °C for 24 h. The starch gel formed was used for the batch wise adsorption of the CGTase. A slurry (15 ml) was prepared by dilution of the starch gel with CGTase buffer (1:2). The mixture was centrifuged, the supernatant was discarded and the extracellular fraction obtained after the expression of the DacD-CGTase (100 ml) was added and mixed. After 20 min at 4 °C, the mixture was centrifuged at 1500×*g* for 3 min and the supernatant was removed. Two washing steps with CGTase buffer (45 ml) were performed. The CGTase was eluted by adding 45 ml of CGTase buffer containing 0.05 g maltose and 0.6 g KCl to the starch and incubated at 50 °C for 1.5 h. The mixture was centrifuged at 9000×*g* for 10 min at room temperature. The supernatant was removed and further centrifuged at 15,000×*g* for 15 min followed by filtration with a 0.22 µm filter. The eluate was concentrated using an ultrafiltration tube with a molecular weight cut-off of 50 kDa (Merck Millipore, Darmstadt, Germany) to a final volume of 1.5 ml.

The purification of the CGTase from the intracellular fractions obtained with the *pelB* construct was performed with 15 ml crude extract obtained from 1 l of culture. Cells were harvested and resuspended in 10 ml CGTase buffer. Cell disruption was accomplished by five cycles of ultrasonic treatment (Sonoplus HD 2200, tip KE 76, Bandelin Electronic, Germany, Berlin). The lysate was centrifuged at 13,000×*g* for 30 min at 4 °C and the supernatant was used as crude extract for purification as described above for the extracellular fraction.

### CGTase purification by immobilized metal affinity chromatography (IMAC)

A 1 ml HisTrap FF column (GE Healthcare, Freiburg, Germany) was used for the purification of the CGTase with a Äkta Pure chromatography system (GE Healthcare, Freiburg, Germany). Buffer A consisted of 20 mM sodium phosphate buffer, pH 7.4 and 500 mM NaCl and buffer B consisted of buffer A with additional 250 mM of imidazole. Equilibration was performed with 5 column volumes of 96% buffer A and 4% buffer B. Extracellular supernatant (100 ml) was loaded onto the column, followed by a washing step of 2.5 CV of 96% buffer A and 4% buffer B. Elution of the CGTase was performed using a gradient from 4% buffer B to 100% buffer B within five column volumes. Fractions of 0.5 ml were collected.

### SDS-PAGE

Protein samples were analyzed using 12% polyacrylamide gels with 4% stacking gel [24]. Pierce Unstained Protein MW Marker was used (Thermo Scientific, Schwerte, Germany). Extracellular fractions were precipitated by 10% (v/v) trichloroacetic acid and concentrated approximately tenfold [25].

## Results and discussion

### Recombinant expression of the CGTase

**Fig. 1:**
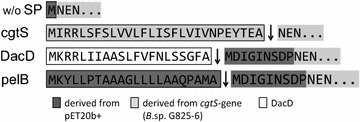
Translated N-terminal sequences of the recombinant CGTase constructs. *Arrows* indicate the predicted cleavage sites

The codon-optimized *cgtS* gene encoding the *Bacillus* sp. G825-6 CGTase were expressed with the PelB, DacD, and the native CgtS SP in *E. coli* BL21(DE3) harboring a pET20b(+) vector (Fig. 1).

**Fig. 2:**
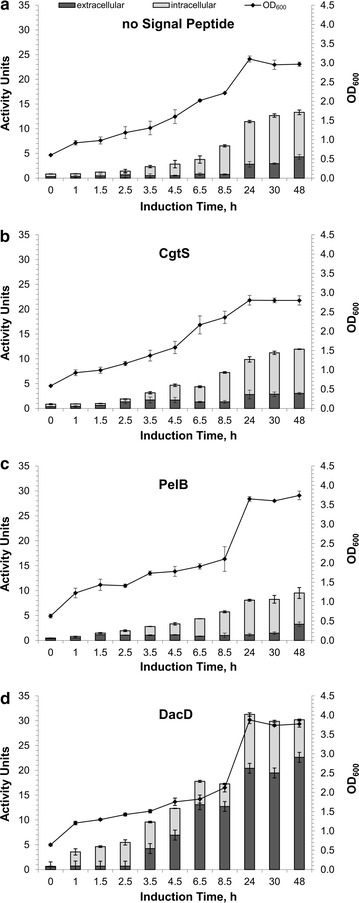
Cell densities of *E. coli* BL21(DE3) and the yield of recombinant CGTase using different SP. **a** CGTase gene without SP; **b** CgtS-CGTase; **c** PelB-CGTase; **d** DacD-CGTase. *Error bars* represent the standard deviation based on triplicate determinations

Figure 2 shows the growth curves of *E. coli* BL21(DE3) harboring the four different plasmids and the corresponding starch-degrading activity in the extra- and intracellular fractions of the cultures. The constructs containing the DacD SP produced an approximately 3.9, 3.2, and 2.7-fold higher activity than those containing PelB, CgtS, and the construct without a SP, respectively. DacD also caused a pronounced secretion of the enzymatic activity into the extracellular medium. After 24 h, 65% of the total enzyme activity was found in the extracellular space. With DacD, a 2.7- to 4.6-fold higher amount of extracellular enzyme was obtained than with the other SP investigated. These results support a previous analysis of the membrane permeability in *E. coli* expressing CGTase which has shown that the recombinant protein accumulated near the inner membrane and blocked the translocation channels [26]. The formation of inclusion bodies is a well-known problem limiting the overexpression of proteins in *E. coli* [27]. Presumably, an efficient translocation of the recombinant protein from the cytoplasm into the periplasm could reduce the formation of inclusion bodies inside the *E. coli* cells. The translocation into the periplasm is therefore a key factor for the further undirected transport of the recombinant protein into the culture medium. Furthermore, the secretion of recombinant proteins into the extracellular medium greatly simplifies the downstream process by avoiding the disruption of host cells resulting in the release of large amounts of intracellular proteins.

An extended induction time up to 48 h did not result in a further increase in the cell density of the constructs and only a small change of the extracellular activity was detected after 24 h.

**Fig. 3:**
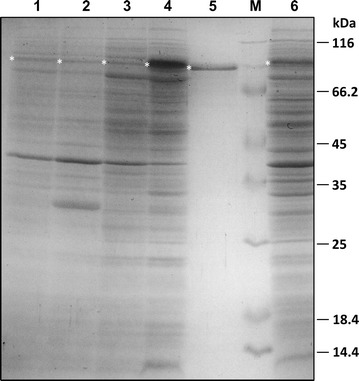
SDS-PAGE analysis of proteins in the extracellular fractions of cultures expressing CGTase genes with different SP. *Lane 1* CGTase without SP; *lane 2* CgtS-CGTase; *lane 3* PelB-CGTase; *lane 4* DacD-CGTase; *lane 5* DacD-CGTase purified by batchwise starch adsorption; *lane 6*: DacD-CGTase obtained with 100 mM glycine added to the culture medium. *M* molecular mass marker. 10 µg protein for extracellular fractions and 1 µg of purified protein was loaded. CGTase bands are marked with an *asterisk*

SDS-PAGE analysis of the extracellular proteins confirmed the superior expression performance of the recombinant CGTase using the DacD SP (Fig. 3). These results confirmed DacD as a suitable general secretory (Sec) pathway-dependent SP for the efficient extracellular production of the recombinant CGTase in *E. coli* BL21(DE3).

### Comparison of DacD with other CGTase-expressing SP

Previous studies have shown that other Sec-dependent SP including PelB and OmpA promoted the transport of recombinant CGTase into the periplasm [15, 28]. However, PelB in our study did not favor a secretion of the CGTase into the extracellular space. By using the native SP of *Bacillus* sp. G1 CGTase for the recombinant expression in *E. coli,* 62.3% of the total activity has been detected in the extracellular medium [13]. In contrast, a yield of only 13.2% of the total activity was found in the extracellular medium using the CgtS SP in this study indicating that the SP for an optimal recombinant production of a specific protein cannot be predicted and requires a screening for suitable candidates. While cleavage site prediction is possible to a certain degree [29], the cleavage site of a distinct protein can change from its natural state when expressed with heterogeneous SP [30]. More precise prediction methods have not been developed yet and available models are lacking a profound understanding of the effects of SP on the translocation process.

### Effect of glycine on the extracellular expression of DacD-CGTase

The addition of 100 mM glycine immediately after the induction with IPTG resulted in a strong increase in the protein concentration in the extracellular fraction. SDS-PAGE analysis of the extracellular proteins obtained from cultures treated with glycine confirmed the presence of a larger amount of host proteins and a lower proportion of the CGTase suggesting an enhanced cell permeability or even cell lysis of *E. coli* and the corresponding release of host proteins in the presence of glycine (Fig. 3) [15, 17].

### Purification of the recombinant CGTase by starch adsorption and by IMAC

Soluble starch pretreated to form a gel was used to purify the recombinant DacD-CGTase found in the intra- and extracellular fractions by batch wise adsorption. This low-cost and rapid method has previously been described for the purification of other CGTases [31, 32].

As shown in Table 1, 13.1% of the total activity was recovered from the intracellular fraction with a specific activity of 1505 U/mg. In contrast, 46.5% of the total enzyme activity could be recovered from the extracellular fraction indicating that the large amount of host proteins in the intracellular fraction had a negative influence on the adsorption of the CGTase to the starch gel (Table 1a). After a 55-fold concentration of the eluate, a specific activity of 1636.9 U/mg of the purified DacD-CGTase from the extracellular fraction was obtained (Table 1b). For comparison, the recombinant DacD-CGTase was also purified by IMAC. The CGTase in the intracellular fraction could not be separated using this method suggesting that the C-terminal His_6_-tag did not properly bind to the Ni-Sepharose 6 matrix. As shown in Table 1c, most of the CGTase did also not bind to the column in the absence of *E. coli* host proteins. Since several intracellular proteins from *E. coli* have shown a high affinity to Ni^2+^, further purification steps, e.g. size exclusion chromatography, would be necessary to obtain a homogenous recombinant protein preparation [33]. From the extracellular fractions, only a low yield of CGTase (2.9%) with a specific activity of 595.6 U/mg could be obtained by IMAC (Table 1c). In contrast, by batch wise starch adsorption, highly pure CGTase preparations could be obtained quickly in high yields without the use of expensive IMAC media.

**Table 1:** Comparison of the purification of extra- and intracellular CGTases by starch adsorption and by affinity chromatography with Ni-Sepharose 6

Fraction	Volume (ml)	Protein conc. (mg/ml)	Total protein (mg)	Volume activity (U/ml)	Total activity (U)	Specific activity (U/mg)	Yield (%)	Purification (-fold)
(a) Purification of the intracellular PelB-CGTase by batch wise starch adsorption								
Crude extract	15	24.7	370.5	330	4950	13.4	100	1.0
Washing step	30	2.1	63.9	37.5	1123.8	17.6	22.7	1.3
Eluate	2	0.22	0.43	324	648	1505.0	13.1	112.6
(b) Purification of the extracellular DacD-CGTase by batch wise starch adsorption								
Crude extract	100	0.079	7.94	14.4	1436.9	181.1	100	1.0
Washing step	90	0.006	0.58	2.7	239.3	411.7	16.7	2.3
Eluate	1.5	0.272	0.41	445.4	668.0	1636.9	46.5	9.0
(c) Purification of the extracellular DacD-CGTase by affinity chromatography with Ni-Sepharose 6								
Crude extract	100	0.079	7.94	14.4	1436.9	181.1	100	1.0
Unbound	100	0.072	7.20	9.9	988.8	137.4	68.8	0.8
Washing step	2.5	0.043	0.11	13.5	33.8	313.3	2.4	1.7
Eluate	3.5	0.020	0.07	11.7	41.0	595.6	2.9	3.3

## Conclusions

The DacD SP strongly promoted the extracellular production of CGTase in *E. coli* and enabled an up to 3.3-fold increase in the yield of total activity compared to the other SP. Furthermore, DacD SP caused a significantly enhanced secretion with a more than 13-fold higher specific activity in the extracellular fraction than in the crude cell extract. The extracellular CGTase could be rapidly purified by batch wise starch adsorption with high specific activity and yield. By using these methods, multiple samples can be purified simultaneously and the purification protocol can easily be upscaled to allow for the screening of libraries of CGTase variants obtained by random mutagenesis experiments. A high-level expression system for the extracellular production of CGTases facilitates the manufacture of industrially relevant CD at lower costs opening up new markets for these inclusion complex-forming oligosaccharides.
